# A Review of the Presence of SARS-CoV-2 in Wastewater: Transmission Risks in Mexico

**DOI:** 10.3390/ijerph19148354

**Published:** 2022-07-08

**Authors:** Mayerlin Sandoval Herazo, Graciela Nani, Florentina Zurita, Carlos Nakase, Sergio Zamora, Luis Carlos Sandoval Herazo, Erick Arturo Betanzo-Torres

**Affiliations:** 1 Department of Engineering in Business Management, Tecnológico Nacional de México/Instituto Tecnológico de Misantla, Misantla 93821, Veracruz, Mexico; mayerli.sandoval24@gmail.com (M.S.H.); genanir@itsm.edu.mx (G.N.); 2 Wetland and Environmental Sustainability Laboratory, Division of Postgraduate Studies and Research, Tecnológico Nacional de México/Instituto Tecnológico de Misantla, Misantla 93821, Veracruz, Mexico; 3 Research Center in Environmental Quality, Centro Universitario de la Ciénega, Universidad de Guadalajara, Av. Universidad 1115, Ocotlán 4782, Jalisco, Mexico; fzurita2001@yahoo.com; 4 Public Works Department, University of Local Government of Martínez de la Torre, Veracruz 93605, Veracruz, Mexico; cynakaser@itsm.edu.mx; 5 Faculty of Engineering, Construction and Habitation, Universidad Veracruzana, Bv. Adolfo Ruíz Cortines 455, Costa Verde, Boca del Rio 94294, Veracruz, Mexico; szamora@uv.mx; 6 Estancia Postdoctoral CONACYT (Consejo Nacional de Ciencia y Tecnologia) Tecnológico Nacional de México Campus Misantla, Misantla 93821, Veracruz, Mexico

**Keywords:** municipal wastewater, virus transmission, COVID-19, epidemiology of wastewater, risks of transmission

## Abstract

The appearance of SARS-CoV-2 represented a new health threat to humanity and affected millions of people; the transmission of this virus occurs through different routes, and one of them recently under debate in the international community is its possible incorporation and spread by sewage. Therefore, the present work’s research objectives are to review the presence of SARS-CoV-2 in wastewater throughout the world and to analyze the coverage of wastewater treatment in Mexico to determine if there is a correlation between the positive cases of COVID-19 and the percentages of treated wastewater in Mexico as well as to investigate the evidence of possible transmission by aerosol sand untreated wastewater. Methodologically, a quick search of scientific literature was performed to identify evidence the presence of SARS-CoV-2 RNA (ribonucleic acid) in wastewater in four international databases. The statistical information of the positive cases of COVID-19 was obtained from data from the Health Secretary of the Mexican Government and the Johns Hopkins Coronavirus Resource Center. The information from the wastewater treatment plants in Mexico was obtained from official information of the National Water Commission of Mexico. The results showed sufficient evidence that SARS-CoV-2 remains alive in municipal wastewater in Mexico. Our analysis indicates that there is a low but significant correlation between the percentage of treated water and positive cases of coronavirus r = −0.385, with IC (95%) = (−0.647, −0.042) and *p* = 0.030; this result should be taken with caution because wastewater is not a transmission mechanism, but this finding is useful to highlight the need to increase the percentage of treated wastewater and to do it efficiently. In conclusions, the virus is present in untreated wastewater, and the early detection of SAR-CoV-2 could serve as a bioindicator method of the presence of the virus. This could be of great help to establish surveillance measures by zones to take preventive actions, which to date have not been considered by the Mexican health authorities. Unfortunately, wastewater treatment systems in Mexico are very fragile, and coverage is limited to urban areas and non-existent in rural areas. Furthermore, although the probability of contagion is relatively low, it can be a risk for wastewater treatment plant workers and people who are close to them.

## 1. Introduction

The appearance of the SARS-CoV-2 (severe acute respiratory syndrome coronavirus 2), which gives rise to the COVID-19 disease, has represented a new threat of infection for mankind. The means of propagation of the virus occurs by different means, and one in discussion is its incorporation in wastewater. The pandemic that initially emerged in China [1,2] and later spread to all the world’s continents should put the sanitation systems of all countries on alert. This is due to some findings such as those of [3], who found the presence of SARS-CoV-2 RNA (ribonucleic acid) in wastewater with a concentration of 10^4^ GC/100 mL, so the authors estimated a removal of 1 to 2 log^10^ during wastewater treatment. Moreover, Ref. [4] previously demonstrated that with SARS (severe acute respiratory syndrome), droplets of liquid contaminated with feces are a potential vehicle for the spread of a respiratory virus to large numbers of people and concluded that coronaviruses can remain infectious for long periods in water.

This situation is of concern in Latin American countries, where the level of sanitation is low, and better management of water resources is required [5]. In the specific case of Mexico, the final disposal of the different types of untreated wastewater, including those of hospital origin, is not clearly known. This represents a situation of alert and concern given that Mexico has been one of the Latin American countries with the highest number of cases of infection by COVID-19 with 5,455,237. It is also alarming that Mexico has the highest mortality rate in the world at 5.81%. The Panamerican Health Organization (PAHO) assured that “Mexico is far from a drastic reduction in cases since in the last week of February this year it reported a 70 percent increase in the number of new infections” by COVID-19 [6] (p. 1).

On the other hand, there is information on studies indicating the presence of SARS-CoV-2 in wastewater, and these are also scarce in Mexico. However, emerging pathogens can enter the wastewater system due to the dissemination of human waste and sanitary material from hospitals. In general, hospitals generate significant volumes of wastewater, ranging from 100 to 1200 L person/day, loaded with toxic chemical compounds, drugs, microorganisms, radioactive elements and radioisotopes, heavy metals, and organo-halogen compounds [6]. These effluents can reach surface water bodies, so it is a situation that puts public health at risk, mainly in areas where there is no wastewater treatment [7].

The above information makes us infer that the probability of the presence of SARS-CoV-2 in wastewater in Mexico is high. Given the low sanitation coverage and low efficiency of municipal wastewater treatment plants (MWWTP) in various regions of the country [6,7,8], SARS-CoV-2 may end up in water bodies with which the population and workers of MWWTPs may have contact.

Due to the above, the present work addresses the following research objectives: to carry out a review on the presence of SARS-CoV-2 in wastewater throughout the world and to analyze the coverage of wastewater treatment in Mexico to determine if there is a correlation between the positive cases of COVID-19 and the percentages of treated wastewater in Mexico and, on the other hand, to investigate the evidence of possible transmission by aerosols and untreated water deposited in water bodies.

## 2. Materials and Methods

**Figure 1:**
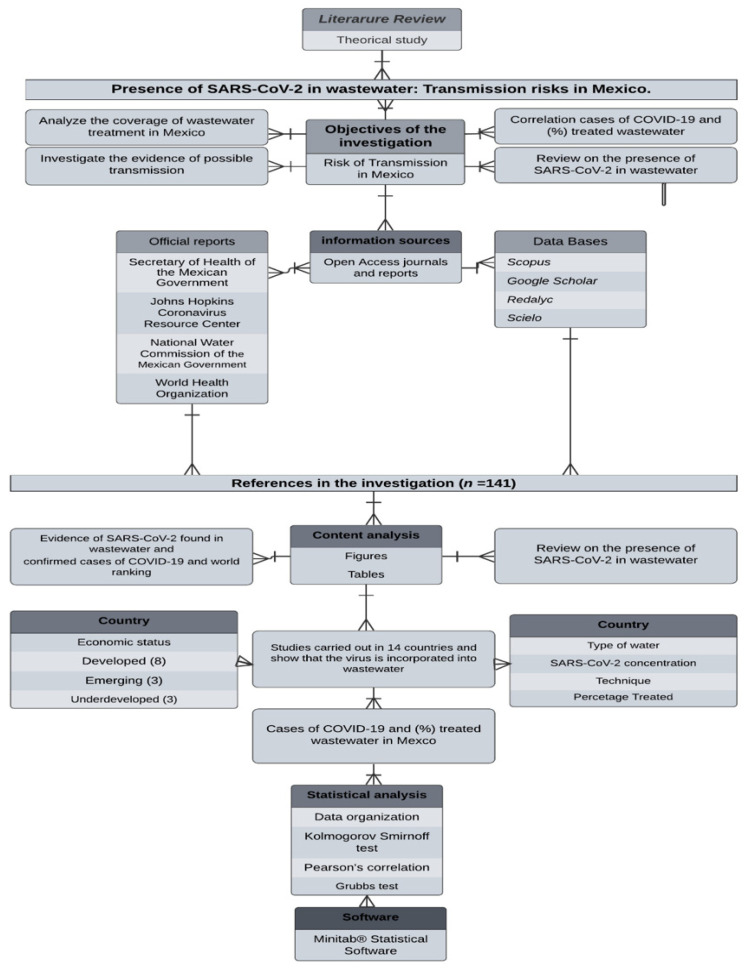
Flowchart of the methodology used in the research.

Relevant literature papers were considered, such as peer-reviewed papers and gray literature from company websites, government portals, and other regulatory agencies, to complement published studies on COVID-19 and wastewater. To perform this review, *Google Scholar, Scopus, Redalyc, Scielo,* and open access journals were used as well as official documents from the World Health Organization, Secretariat of Public Health of the Mexican Government, and the Johns Hopkins Coronavirus Resource Center [9,10] for the period from February 2020 to May 2021. Finally, the information from the WWTP in Mexico was used using the official information from the National Water Commission dependent on the Government of Mexico. Additionally, the methodology followed in this study is described in Figure 1.

### Statistical Analysis

Statistically, a Pearson’s correlation was performed to determine if there is a correlation between positive cases of COVID-19 in Mexico and the percentage of treated wastewater, with a confidence level of 95%, was previously done Kolmogorov–Smirnoff normality test, and subsequently the Grubbs test was subsequently applied to show evidence of these atypical values in the data using Minitab^®^ Statistical Software (State College, PA, USA).

This study was performed with available data from the Secretariat of Public Health on 11 April 2021 from 2019-COVID-NET [11], and the National Water Commission [12]. Figure 1 shows the methodology used in the research.

## 3. Results and Discussion

### 3.1. Presence of SARs-CoV-2 in Municipal Wastewater

The World Health Organization [13] states that a person can contract COVID-19 through contact with another person infected with the virus. This is considered the main transmission mechanism that occurs through the droplets that come out of the nose or mouth of an infected person when coughing, sneezing, or talking. These drops are relatively heavy, so the distance they reach is not very long, and they fall quickly [14]. However, a person can also contract the virus without inhaling the droplet from an infected person, and studies have shown possible transmission through inhalation of infectious aerosols and reuse of untreated or partially treated sewage, as the virus can survive in sewage and aerosols for a long time. Therefore, in addition to close contact, spread through contaminated surfaces and airborne transmission through aerosols can also occur [15].

This airborne route involves much smaller droplets that can float and move long distances on air currents [16]. On the other hand, [17] states that viral replication appears to take place in the mucosal epithelium of the upper respiratory tract, and then, further multiplication occurs in the lower respiratory tract and gastrointestinal mucosa; thus, non-respiratory symptoms such as headache, diarrhea, and conjunctivitis have been found [18]. From the gastrointestinal symptoms, it is possible to infer that the virus can be spread through feces.

SARS-CoV-2 reaches wastewater in two main ways: The first is through the feces of the carriers, from homes or health institutions, as the virus has been found in human feces up to 33 days after the patient tested negative for COVID-19 [6,19]. The second way is through the final disposal and cleaning of materials and equipment used for the care of people infected with COVID-19. The presence of SARS-CoV-2 in wastewater represents a possible risk for the rural population since surface and groundwater without treatment are used as drinking water. This becomes a direct impact on public health [20] since viruses can be present in waters or other surfaces in contact with feces, from which potential vector insects could spread SARS-CoV-2 [21,22].

During the SARS-CoV outbreak in 2003, SARS-CoV RNA was found in the sewage treatment facilities of two hospitals in Beijing, China, where infected patients were treated [23]. In the case of SARS-CoV-2, its presence has been reported in hospital wastewater [24] and community sewage collection stations [25,26]. For example, in Paris, France, a study of raw sewage found positive results for final SARS-CoV2 samples. It was also confirmed that the increase in genome units in raw sewage accurately followed the increase in the number of fatal cases observed at the regional and national levels [27]. In Ecuador, the presence of the SARS-CoV-2 virus was found in samples of domestic wastewater collected from the lagoon systems of Punta Carnero and Playas [28].

Table 1 shows a rapid literature review of the studies that have been carried out in the world on the presence of SARS-CoV-2 in wastewater. The results correspond to studies carried out in 14 countries and show that the virus is present in wastewater. This occurs in developed, developing, and emerging countries. This means that if wastewater treatment is poor, the virus will enter surface waters that function as receiving water bodies. In this sense, in developing and emerging countries, improvements in wastewater treatment plants are needed to prevent the virus from reaching surface waters [29].

Table 2 classifies the countries with confirmed cases of COVID-19 where there is evidence of the presence of SARS-CoV-2 in wastewater; the highlight of this classification is that regardless of the economic status of the country, the resources available for the treatment of its wastewater, technology, and treatment coverage, the data suggest a possible deficiency in the operation of the WWTP, which cannot eliminate SARS-CoV-2 RNA with conventional methods, an aspect that is alarming and shows poor treatment methods since normally, these plants should have disinfection stages capable of eliminating the virus prior to reuse. This problem opens an opportunity to develop alternative methods for the elimination of the virus in the WWTP and, failing that, to strengthen the disinfection systems that guarantee its elimination.

### 3.2. Potential Risks from Wastewater Management

The presence of infectious coronavirus particles in wastewater can cause health problems for people exposed to wastewater [18]. Approximately 1.8 billion people worldwide use water contaminated with feces as drinking water; if proper precautions are not taken, the risk of spreading COVID-19 can increase by several times [45]. In addition, the presence of urban flooding and sewage overflow during the rainy season in different latitudes may increase the risks of virus spread in areas and communities affected by COVID-19 [46]. Another source of concern is overcrowded human settlements, which can become an environment conducive to the spread of the virus [47].

On the other hand, wastewater reuse to recover water, nutrients, and/or energy has become an important strategy, especially in water-scarce areas; biosolids are by-products of the wastewater treatment process and contain a large amount of nutrients that are used as organic fertilizers in agriculture and forestry [48,49]. However, the presence of SARS-CoV-2 and other pathogens in these wastes requires careful handling, and this applies also for waste materials produced at different stages of wastewater treatment plants, including application of manures and biosolids to improve soil quality as a well-known method agricultural practice, for chicken feed, and for lake restoration among others [50,51,52,53]. It is important to note that animal-related coronaviruses have been shown to persist in lake water and pasteurized wastewater; they are contagious and last from a few days to weeks [4,54].

Available information on virus survival indicates that the population most at risk is those exposed to untreated sewage; these people may include sewage treatment plant workers and the general population who may come into direct contact with sewage through faulty pipes or sewage networks [55,56]. In wastewater treatment plants, inhalation of aerosols or droplets contaminated by infectious virus particles is reported to be the main route of the spread of the coronavirus [55,57,58,59,60]. However, some studies have considered the risks for workers in wastewater treatment plants; therefore, there is a lack of information on possible infections from such exposure [55].

Ref. [56] (p. 7) reported that “During aggressive outbreak conditions when 3% of the population served by the WWTP is infected, risk profiles are notably higher with up to 14 cases of illness predicted among 100 WWTP operators accidentally exposed to SARS-CoV-2 in raw sewage, by inhalation”; on the other hand, it applied an exposure scenario assuming that WWTP operators accidentally ingest 1 mL^−1^ of raw wastewater containing SARS-CoV-2 through the mouth while performing routine activities.

By contrast, Ref. [60] (p. 5) stated that “the highest risk of exposure is related to spreading and handling untreated feces, followed by untreated municipal sludge, class B biosolids, while the lowest risk is associated with spreading or handling class B biosolids and recommend that workers continue to follow industry safety practices to minimize risk”. Despite previously described research, Ref. [61] confirmed that, under laboratory conditions, infectious SARS-CoV-2 was detected in aerosol for a maximum of 16 h, an aspect that opens up a possibility for future research on a larger scale in WWTPs in the field; since it is a review carried out by [62], no convincing evidence was found in China, Spain, and Italy of virus infectivity in wastewater.

### 3.3. Wastewater Treatment in Mexico and SARS-CoV-2 Risks

In Mexico, there have been great advances in sanitation, according to official information: in 2017 there were 2536 municipal wastewater treatment plants that treated 123.6 m^3^/s of wastewater and 3025 industrial wastewater treatment plants with a capacity of 75.9 m^3^/s [8], a figure that decreased for 2018 in 10 municipal wastewater treatment plants and in 16 industrial wastewater plants [12]. This reduction in the number of treatment plants contributes significantly to an increase in pollution in water bodies such as rivers, lakes, and seas that receive wastewater without treatment. Regarding hospital effluents, as far as is known, they are not treated separately but are incorporated into sewage systems, which increases the biological risk of municipal wastewater [6,63,64].

However, data on wastewater treatment in Mexico are contrasting: on the one hand, progress in sanitation is observed in the country, supported by a robust National Water Law [65] that has been part of the agenda of the governors in recent years and has allowed growth from 30.55 m^3^/s treated in 1992 [66] to 135.6 m^3^/s in 2018 [8] in the treatment of municipal wastewater (increased by 444% for that period). This has made Mexico the Latin American country with the highest growth in wastewater treatment [67]. However, according to official data, only 63% of municipal wastewater is treated in the country [68] and 33% of industrial wastewater [69]. Therefore, it is still required to increase the volume of treated wastewater. Still, wastewater treatment in rural populations is very limited.

**Figure 2:**
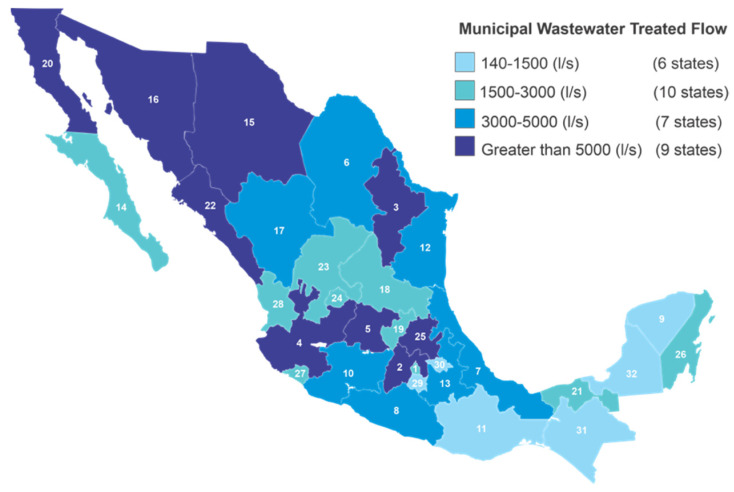
Treated municipal wastewater flow rate by 32 states in Mexico based on CONAGUA data [8] and the National inventory of municipal drinking water and wastewater treatment plants in operation [70].

On the other hand, in Figure 2, according to the National Water Commission, nine entities generate a flow rate greater than 5000 L/s of municipal wastewater; four of them are located in the central part of the country (State of Mexico, Guanajuato, Jalisco, Hidalgo) and five in the northern zone (Nuevo León, Chihuahua, Sonora, Baja California, and Sinaloa), with an average treated flow rate of 78. 16%; the State of Mexico is the state that least treats its wastewater (65.68%), and Hidalgo is the one with the highest flow rate treated (92.89%) [8].

In the range of flow generated from 3000 to 5000 L/s are seven states, three located in the northern zone (Durango, Coahuila, and Tamaulipas) and four in the central part (Veracruz, Guerrero, Puebla, and Michoacán), with an average treated flow of 77.31%; Tamaulipas only treats 55.59% of its municipal wastewater. With a generated flow rate between 1500 to 3000 L/s are 10 federative entities that are scattered within the country (Aguascalientes, Tabasco, Nayarit, Mexico City, San Luis Potosí, Querétaro, Quintana Roo, Colima, Baja California Sur, and Zacatecas) with an average treated flow of 72. 32%; in this case, Mexico City is the one that treats the lowest percentage of wastewater (not only in the area but also within the entire Mexican Republic) with 43.74%m and Tabasco is the entity that treats the highest percentage (89.73%) [8].

Finally, there are six states (Chiapas, Oaxaca, Morelos, Tlaxcala, Yucatán, and Campeche) that have a flow rate lower than 1500 L/s, with an average treated flow of 66.47%. In this area, the state that least treats wastewater is Morelos (46.08%), and the one that treats the most is Campeche (92.13%).

### 3.4. Is It Possible to Find a Relationship between COVID-19-Positive Cases and the Level of Wastewater Treatment in Mexico?

After China, Mexico is the country that uses the most wastewater for agricultural purposes [71]. This is worrying since current treatment methods do not guarantee the elimination of microorganisms, such as viruses and parasites [72]. Wastewater, if not treated properly, endangers the environment and human beings since pollutants can infiltrate aquifers or become incorporated into soils [6]. Table 3 shows the data on treated water in Mexico versus cases of COVID-19.

**Figure 3:**
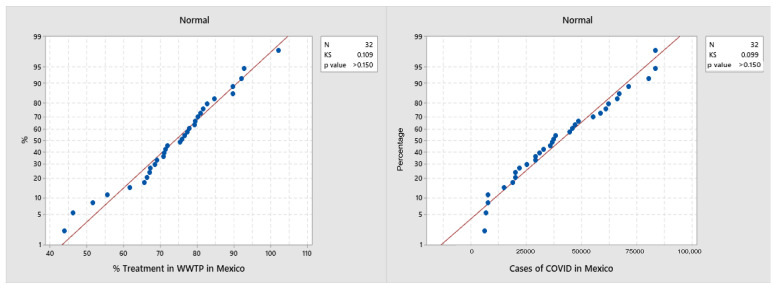
Kolmogorov–Smirnov normality test chart.

On the other hand, when analyzing the percentage of treated wastewater (%TWW) by state throughout the country, a national average of 73.9% was found. Regarding the correlation analysis between the % TWW and the accumulated positive cases of COVID-19, the Kolmogorov–Smirnoff test was applied, and it was observed that the variables fit a normal distribution (Figure 3). Therefore, a Pearson’s correlation analysis was performed.

**Figure 4:**
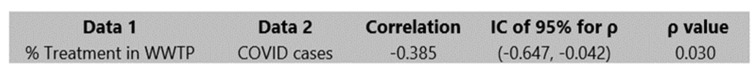
Correlation results between positive cases of COVID-19 and percentage of treated wastewater in Mexico, a significant negative correlation (r = −0.385) was found between both variables (*p* = 0.030).

Regarding the correlation analysis, a significant negative correlation r = −0.385 was found between both variables (*p*−value= 0.030); that is, the higher the treated wastewater flow, the more the positive cases of SARS-CoV-2 virus tend to decrease; the confidence intervals (CI = −0.647, −0.042) indicate a range of probable values for the correlation coefficients with a 95% probability that the data analyzed are within this interval, which is observed in Figure 4.

The *p*-value is key to determining if the correlation coefficient is statistically significant. In this way, to determine if the correlation coefficient is statistically significant, the *p*-value was compared with the level of significance α = 0.05, and the *p*-value ≤ α indicates that the correlation between the means is statistically significant; therefore, it can be concluded that the correlation is statistically significant.

**Figure 5:**
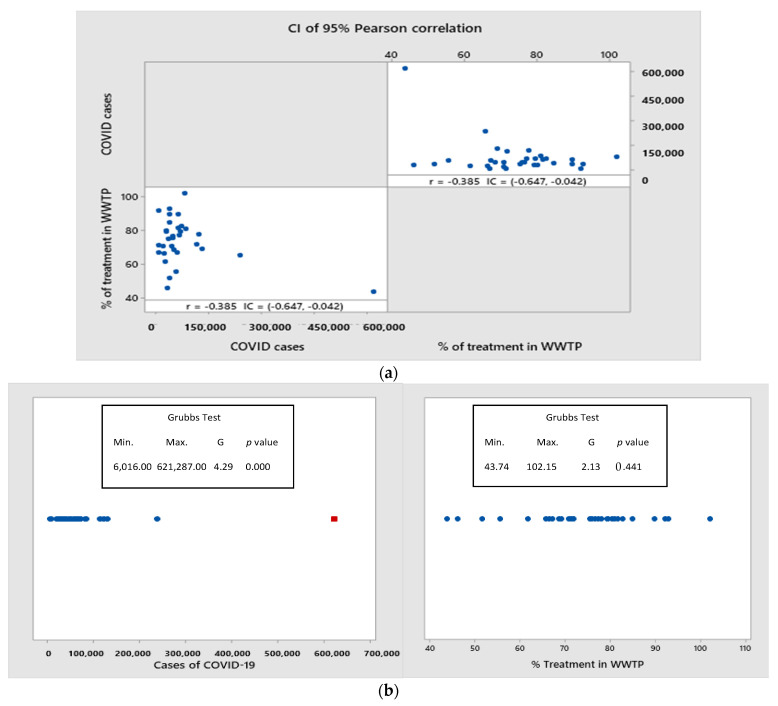
(**a**) Graphic correlation between positive cases of COVID-19 and percentage of treated wastewater in Mexico. (**b**) Grubbs test to determine outliers of positive cases of COVID-19 and percentage of treated wastewater in Mexico.

However, when observing Figure 5a, the correlation effect seems to be due to the atypical values in Mexico City and the state of Mexico, which are the states with the highest number of accumulated cases. Therefore, the correlation found should not be taken conclusively on a cause–effect relationship between the evaluated parameters but rather as a suggestion that the level of sanitation should be considered as one more factor to avoid the spread of SARS-CoV-2.

It is important to mention that the ecological correlation shown between wastewater treatment and COVID-19 cases may be affected by biases due to the spatial and temporal variability of the infection and relationships with other factors that affect the behavior of the virus, such as the population density of the different states of the republic, the climate, and those physical, chemical, and biological aspects that can influence the persistence of viral RNA in wastewater. These factors include temperature, sunlight, ionic strength, presence of antiviral chemicals, solids content, residence time in sewer, and microbial antagonism [71,72,73]. 

Regarding the correlations in Mexico, they are different from in other countries, where the viral concentrations showed good correlations with the number of cases of COVID-19 in the community, which indicates that it is possible to apply statistical tools to predict future outbreaks, the presence of SARS-CoV-2 that would be useful and provide early evidence that the virus circulates in a certain geographical area [62,74,75,76]. The studies demonstrated that the quantitative levels of viral RNA in wastewater are related to the number of COVID-19 cases.

In our case, since it is a national study, it was determined to apply the Grubbs test to show evidence of these atypical values in the data of positive cases of COVID-19 in Mexico, and from the confirmed COVID-19 infections, it is evident that for the largest cities and the most densely populated, the data indicated outliers in contrast to the states of the republic with less population density (Figure 5b). This test is based on the null hypothesis, namely that all data values come from the same normal population, and the alternate hypothesis that the largest data value is an outlier, with a significance level α = 0.05.

### 3.5. How to Reduce the Risk from Wastewater

Faced with poor wastewater management, the widespread transmission of COVID-19 can occur with a low probability due to community interactions, especially in low-income countries where many households share water and sanitation systems [77].

In WWTPs, the elimination of SARS-CoV-2 is possible and the mechanisms have been found to include virus adsorption on larger aggregated particles that are separated from wastewater by sedimentation [78], retention by membranes and biofilm layers, predation and enzymatic degradation in membrane bioreactors [79], and inactivation by disinfection processes such as ultraviolet lamps (UV) [80,81,82] and chlorination and ozonation [83]. As already mentioned, unfortunately, a large percentage of wastewater does not go through a treatment system such as those described above.

On the other hand, it is important to strengthen conventional water treatment methods that use filtration and disinfection, such as those of municipal water treatment plants, to eliminate or inactivate SARS-CoV-2 particles through the disinfection of water with chlorine, which ensures an adequate level of protection for drinking water. SARS-CoV-2 can be inactivated by free chlorine with a concentration greater than 0.5 mg/L and by chlorine dioxide with a concentration greater than 2.19 mg/L [23]. Ref. [54] stated that SARS-CoV-2 can be effectively inactivated by surface disinfection procedures with 62–71% ethanol, 0.5% hydrogen peroxide, or 0.1% sodium hypochlorite within 1 min.

It is necessary to increase the volume of treated wastewater as well as to treat hospital wastewater separately. Due to the diversity of pollutants in hospital effluents, which include domestic wastewater and medical services [84], its pollution power is much greater than that of municipal wastewater. A hospital with 1000 beds and laundry is as polluting as a city with a population of 10,000 [85]. Therefore, it is essential to prevent these waters from reaching the municipal WWTP.

In addition, it is crucial to train and inform the personnel of municipal WWTPs as well as to provide them with protective equipment (masks and special suits) for the respiratory tract, eyes, and extremities to work with wastewater and avoid further contact.

Numerous diseases can be transmitted through water by different pathogenic organisms (helminths, protozoa, bacteria, and viruses). Among those caused by viruses, the following stand out: hepatitis A and E, gastroenteritis, meningitis, respiratory infections, and adenoviruses [86].

It has been theorized that fecal–airway transmission by inhalation of fecal particles with the presence of viable virus in the form of aerosol droplets [24] is also speculation so far based on the fecal findings of SARS-CoV-2, which requires further confirmation from serious scientific studies. On the other hand, some authors confirmed that SARS-CoV-2 has been found in the urine of COVID-19 patients [87,88].

Ref. [89] stated that although there is no convincing research that affirms of fecal–oral, fecal–nasal, or sewage transmission, they do constitute a potential source of transmission to be investigated and assess risks, so there will be a window for future research with appropriate protocols in this regard. The study of wastewater can establish the basis for the detection of other potential health risks, such as new variants of SARS-CoV-2, as well as Zika virus, norovirus, and others. In addition, it becomes a frontier tool for the management of various pandemics and for efficient and timely crisis management [90].

In the case of SARS-CoV-2, it has been detected in different countries, the first being the Netherlands [25,29] and then in Australia [31], the United States, France, and Italy [41,91] in raw sewage [32] from both high and low virus circulation areas. With the aid of molecular techniques, a concentration of up to 10^6^ copies per liter has been detected in raw water, while in treated wastewater, the figure has been 10^5^ copies per liter [92].

However, through quantitative analysis by polymerase chain reaction (qPCR), it is possible to detect the presence of this genetic content in wastewater and to identify the incidence and prediction of diseases, such as that produced by SARS CoV-2 [38]. This is a tool used in humans, where in wastewater, its use should focus on identifying the appropriate sampling points to have a predictive effect.

Thus, [93] stated that the study and monitoring of SARS-CoV-2 in wastewater are intelligent strategies for the early and massive detection of the virus in addition to being a non-invasive alternative to identify areas and critical points of the epidemic. In a territory, this is an aspect that should be considered for its correct application.

In this way, COVID-19 surveillance system through wastewater analysis could be implemented, also known as wastewater-based epidemiology (WBE), which has already been successfully tested in several countries [94]. It works through a strategy of several phases: (1) design of the sampling plan, defined by the points of greatest relevance or incidence, hospitals and health centers, schools, and entire regions and (2) conducting analyzes by RT-qPCR, whose results can be studied through the use of a digital platform that employs GIS and allows correlating the analytical data with the epidemiological data of virus prevalence in the population [38].

The wastewater study predicts and warns about COVID-19 outbreaks 7 to 10 days earlier than the official registration of cases [95]. Moreover, it identifies cases even before the onset of symptoms, which promotes the implementation of measures specific to the local context with a short response period, limiting the impact of the epidemic on the economy and on the daily lives of citizens.

The study according to [96] was been tested in more than 15 countries around the world, including Mexico; it is possible to state that wastewater analysis will assist in the early detection of outbreaks if used appropriately.

In developing countries, such as Mexico, irregular access to water sanitation hinders the homogeneous implementation of this SARS-CoV-2 monitoring strategy [87].

### 3.6. Can SARS-CoV-2 Survive in the Environment in the Form of Bioaresols?

Thus far, person-to-person transmission of SARS-CoV-2 and direct contact and respiratory tract indirect contact through fomites have been documented [97,98] and possibly by aerosols [88,99,100,101,102,103,104]. Regarding the infectivity of the samples, Ref. [105] showed that SARS-CoV-2 virions remained infectious for up to 16 h in aerosols of respirable size, suggesting that aerosols are likely to be a route of transmission. Regarding the survival of the virus in the environment [106], they reported that SARS-CoV-2 can remain infectious in the environment on a variety of surfaces for several hours or even days:

❖ 4 h on copper surfaces;
❖ 24 h in cardboard;
❖ Two or three days in stainless steel;
❖ Three days in plastics.

All these materials are present in the treatment plant facilities.

Studies reporting on SARS-CoV-2 identified potential methods of transmission through:

❖ Vertical transmission during vaginal delivery [107,108];
❖ Sexual transmission [109,110];
❖ Transmission from domestic cats [111];
❖ By contact with waste generated by individuals affected by COVID-19 [112];
❖ Breastfeeding [113];
❖ The possible spread to new wild hosts, such as bats, mustelids, and sand raccoons [114].

Currently available scientific data seem to indicate that humans infected with SARS-CoV-2 can infect other mammals, including dogs [115], cats [116], and farm-raised mink [117]. However, it remains unclear whether there is a significant risk that these infected mammals will transmit the virus to humans. The latter effect of infected humans and the introduction to the natural habitat by wastewater discharges is plausible since it is common for these types of mammals to come into direct contact with humans and with the receiving bodies of wastewater.

Ref. [118] determined the ability of SARS-CoV-2 to survive living in varied environments, humidity, and temperature influence; the excreted virus leaves a possibility for fecal–oral transmission of the virus [119], while feces act as an important cause of viral genomic units prevailing in wastewater, and survival of SARS-CoV-2 RNA in wastewater is possible for several days [120], thus probing the uncontrolled impact of SARS-CoV-2 on the environment [121]. That is still difficult to determine due to the different geographical conditions and different designs of WWTPs in the world.

This hypothesis is possible to understand because the virus can survive in patient toilets and drains in treatment plants with inappropriate disinfection systems. Ref. [122] stated that there is a very low risk of SARS-CoV-2 concerning effluent that has been treated for non-potable applications, but untreated wastewater can potentially be transmitted by the transmission of the virus to WWTP workers.

Importantly, the excreted virus leaves a possibility for fecal–oral virus transmission [123], while feces act as a major source of viral genomic units that are prevalent in wastewater, and survival of SARS-CoV-2 RNA in wastewater is possible for several days [124].

### 3.7. Is the Virus Present in the Wastewater Infectious Enough to Cause the Disease Regardless of the Means of Transmission?

It is shown that SARS-CoV-2 RNA was consistently excreted in the feces of almost 50% of symptomatic patients with a concentration of 1 × 10^8^ RNA per stool sample [119,120,121,122,123,124,125], and researchers examined the viral load in the feces of COVID-19-positive patients and revealed the presence of SARS-CoV-2 RNA at a concentration level of 5 × 10^3^–5 × 10^7.6^ genome copies/mL; this can be interpreted in that a single person can discard billions of copies of SARS-CoV-2 RNA, thus contaminating wastewater [125]; with this evidence of the presence of viral fragments, it does not necessarily mean that they are infectious, but the need for preventive measures is real and should be justified by the global infectious risk already proven for other pathogens.

It is important to note that SARS-CoV-2 infection by aerosols in wastewater is still under discussion. Although the infectious risk of exposure to wastewater aerosols for other viruses has been demonstrated, this possibility exists, and no finding has yet demonstrated viability of this virus in wastewater. However, open drains for agricultural use, bodies of water that receive untreated and treated wastewater, overflow water from the sewage system, as well as monitoring the management of primary and thickened sludge from treatment plants are potential samples for early detection of SARS-CoV-2 [62].

On the other hand, Ref. [62] considered that the main risk factor in the WWTP is biological aerosols, associated with bioaerosols generated by aeration and the bubbles they generate, dehydration, and the necessary mechanical aeration that generates water and is dissipated into the environment during the operation of a treatment plant.

Exposed the previous method, in contrast to what was exposed in other studies, the QMRA analysis (quantitative microbiological risk assessment) showed a relatively high risk of SARS-CoV-2 infection for wastewater workers through exposure to bioaerosols from the WWTP [126].

### 3.8. Possible Solutions to Minimize the Risk

Ref. [62] commented that the International Summit held by The Water Research Foundation (WRF) in late April 2020 identified four potential use cases for wastewater monitoring data, including:

1. Trends/changes in occurrence;
2. Evaluation of community prevalence;
3. Risk assessment;
4. Viral evolution.

Refs. [127,128] suggested that quantitative microbial risk assessment (QMRA) is a useful tool that has been used to estimate human health risks associated with exposure to pathogens in different environmental matrices and has been applied to assess health risks associated with bioaerosols and sewage [129]. It is important to allocate resources for treatment at the WWTP, for example, for the disinfection process at the final treatment by chlorination, ozonation, or UV disinfection (to protect the receiving water bodies) and membrane technologies for those cases in which the treated wastewater is made drinkable. In addition, it is important to control the human settlements (invasions) that are carried out in the surroundings of the stabilization ponds to avoid contamination with viral particles of SARS-CoV-2 as a preventive measure as well as to avoid the access of mammals (which have been shown to be able to be infected).

Regarding recommendations for WWTP workers, protection is important: minimizing the production of droplets and aerosols of feces and municipal sludge in the collection system and in the head works with systems that agitate the water to a lesser extent during the operation and wearing thick rubber gloves over surgical gloves as well as protective goggles or a face shield are necessary. In these environments where aerosol-producing techniques are performed, personnel must wear an N95, FFP2, or FFP3 protective mask; the World Health Organization has provided guidance on safety at work [130].

The government of Mexico makes available the “Specific technical guidelines for the reopening of economic activities” and guides to develop of health security protocols for micro-, small-, and medium-sized enterprises [127], which includes a list of 56 types of companies, but unfortunately, it does not exist for WWTP, so it is necessary to create a specific one for this purpose; this shows that importance is not given to proper management of wastewater and its value as a means of spreading information to receiving bodies and health care for workers.

There is a need for a standardized method for the detection of SARS-CoV-2 in wastewater to conduct prevalence studies not only in that environment but also in water treatment operations and processes.

The study of wastewater opens a space for its investigation in the new normality and to consider wastewater as a reliable source of information for the surveillance of present and future epidemics and in general of the health of the world’s population. This may be an opportunity to give it the importance it deserves in all countries.

### 3.9. Added Value of this Study

The focus of this work is to estimate these health risks incorporating data from the literature; thus, the epidemiological, clinical, laboratory, and microbiological findings of the presence of SARS-CoV-2 in wastewater were presented. As the investigations carried out to date have confirmed the presence of the new coronavirus in various parts of the world in the WWTP, the correlations suggest a useful tool to determine the presence as a bioindicator that could be called (BIO-COVID-WWTP).

The correlation of the presence of SARS-CoV-2 is complex; taking into consideration the specific number of cases, it is greater when we incorporate percentages of water treated in the treatment plants by locality, an aspect that had not been attempted before. To date, this complication lies in external factors, such as the sampling method and time, dissolutions due to mixing of water and rain, environmental factors (physical, chemical, and biological), and of course inactivation by temperature, pH, organic matter, and the chain of custody coupled with the number of tests in real time, which are each minor. Despite this, an approximation of this low but statistically significant negative correlation was obtained, which allows opening a window of opportunity for future lines of research.

### 3.10. Implications of All the Available Evidence

Although this new coronavirus still has a long way to go to understand its behavior in WWTPs and its possible risk of infection, the possibility of transmission by exposure to bioaerosols to WWTP workers cannot be excluded.

**Figure 6:**
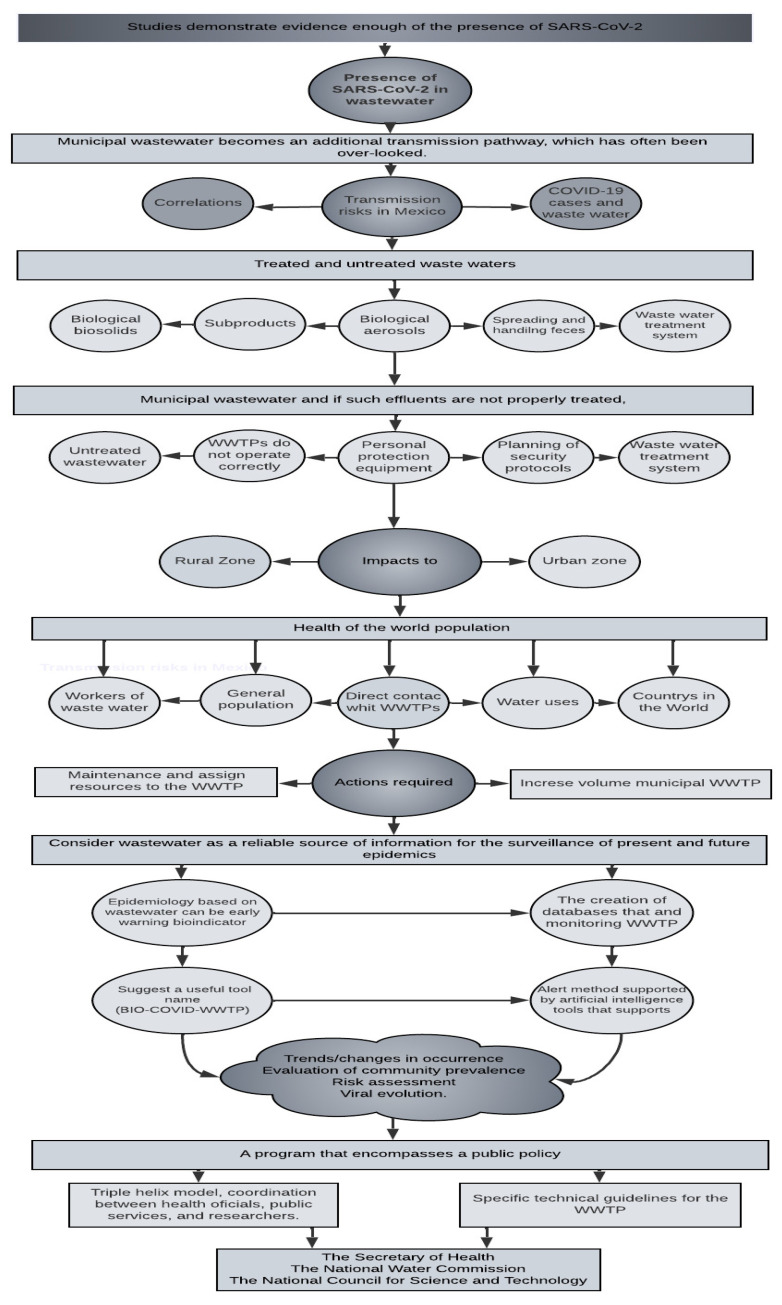
Diagram of relationships within the results of the investigation.

The vigilant epidemiological control of the person responsible for the operation of the WWTP, in this case, the National Water Commission, is important for its investigation and to prevent risks to the health of humans in contact with these treated and untreated waters both in urban and rural areas. Figure 6 shows the diagram of the relationships elucidated in the results of the investigation.

## 4. Conclusions

Based on the objectives of this research, it is possible to conclude the following:

(1) SARS-CoV-2 is present in municipal wastewater, and if such effluents are not properly treated, the virus can reach the receiving aquatic bodies. Thus, municipal wastewater becomes an additional transmission pathway, which has often been overlooked.
(2) In the rapid review of scientific articles, where it was classified by country and the degree of development, to date, nothing similar was found, and this aspect is forceful: “SARS-CoV-2 despite the economic status of the countries, even the virus is incorporated, regardless of the economic potential of the country, where it would be assumed that its treatment systems are robust and modern”.
(3) Although a correlation was found between the variables of %TWW by state and positive cases of COVID-19 in Mexico, the cause–effect relationship should be considered with caution since wastewater is not the main route of transmission of SARS-CoV-2. However, it should serve to emphasize the importance of increasing the level of wastewater treatment to reduce exposure of the population and contamination of drinking water sources.
(4) Further confirmatory studies of fecal–air, fecal–oral, and fecal–nasal transmission or by sewage, by inhalation of fecal particles with the presence of viable viruses in the form of aerosols, and by the presence of the virus in receiving water bodies are still necessary. On the other hand, it is conclusive in the review carried out that these poorly investigated pathways may constitute a potential source of transmission.

## 5. Recommendations

Since in Mexico, a good part of the WWTPs do not operate correctly due to lack of maintenance, in the current pandemic situation, this should be corrected as soon as possible, with emphasis on the revision of the disinfection processes in the final stage.

In WWTPs, the use of protective equipment by the technical personnel who operate the WWTPs is crucial as well as their training and information on the risks involved in handling wastewater and the waste generated.

At the household level, it is suggested to chlorinate the cisterns at least once a month and maintain the amount of free chlorine between 1 and 3 mg/L.

The detection of emerging viruses in wastewater is feasible in the future as an alert method supported by artificial intelligence tools with a program that encompasses a public policy that supports the creation of databases that monitor the existing infrastructure in addition to strengthening current treatment systems.

The research carried out in Mexico that demonstrates the presence of SARS-CoV-2 is scarce; it is still necessary to carry out a greater number of studies in large areas of the country that support defining critical points in the current fourth wave of infections and take preventive measures. Based on the presence of the virus in the WWTPs in the receiving bodies and due to the massive detection tests that have notably decreased in hospital centers, this bio-indicator can be useful to predict new zoned outbreaks and take action before contagions grow.

It is important to consider that the measurement in wastewater aerosols can be useful and informative for risk assessment in treatment plants and thus develop a special protocol for the detection of SARS-CoV-2 in wastewater by area, climate, and by treatment volume of the PATR, which would be useful to implement. 

Wastewater research opens a space for its investigation in the new normality and considers wastewater as a reliable source of information for the surveillance of present and future epidemics and in general of the health of the world population. An opportunity opens up to give it the importance it deserves in all countries. 

Epidemiology based on wastewater can be applied in the future as an early warning bioindicator tool for virus outbreaks and for monitoring, case tracking, and obtaining information at the local, regional, and national scale.

Surveillance of WWTPs requires a triple helix model: coordination between health officials, public services, and researchers. These are the Secretary of Health, the National Water Commission, the Mexican Institute of Water Technology, the National Council for Science and Technology, and other institutions interested in investigating the presence of SARS-Cov-2 in more than 2400 WWTPs in Mexico.

## 6. Future Lines of Research

Future studies should investigate viral infectivity in treated and untreated wastewater in urban and rural areas with a high incidence of SARS-CoV-2 in Latin America where treatment systems are limited and reliability and coverage low.

The survival of SARS-CoV-2 in the components of sanitation systems and, above all, its ability to be transmitted to recipient bodies where there is no treatment should also be studied.

Until now, there are limitations in research that conclusively demonstrate that exposure to wastewater with SARS-CoV-2 has been implicated as a transmission vector, so and addressing this gap in science would support answering the hypothesis about infection by contact with wastewater with SARS-CoV-2 viral RNA.

The limited data available do not clearly answer whether SARS-CoV-2 is infectious in wastewater for humans, but more research is needed to conclude whether or not wastewater is a transmission route for SARS-CoV-2 infection through different pathways: bioaerosols in the treatment plant and discharges to water bodies and throughout the sanitation system from houses, hospitals, sewers, WWTP, and receptor bodies.

High-quality research is urgently needed to clarify the relative importance of the different routes of transmission and the importance of airborne transmission when techniques that minimize the production of aerosols are not put into practice, which seems to be the greatest risk of contagion for WWTP workers.

## Figures and Tables

**Table 1:** Studies carried out in 14 countries and show that the virus is incorporated into wastewater.

Economic Status	Country	Type of Water	Technique	>SARS-CoV-2 Concentration	Reference
Developed	Netherlands	Untreated wastewater	RT-qPCR	26–1800 gc/mL.	[30]
	Germany	Untreated wastewater	RT-qPCR	30.0 and 20.0 gc/mL inflow. 3.0 and 20 gc/mL effluent.	[31]
	United States of America	Untreated wastewater	RT-qPCR	57 to 303 gc/mL.	[27]
	Australia	Untreated wastewater	RT-qPCR	1.9 to 12 gc/100 mL.	[32]
	France	Untreated wastewater	RT-qPCR	10^6^ eq/L gc/L.	[33]
	United Arab Emirates	Wastewater	RT-qPCR	Wastewater influents: 7.50 × 10^2^ and 3.40 × 10^4^ cg/L, Untreated wastewater: 7.50 × 10^2^ to over 3.40 × 10^4^ gc/L	[34]
	China	Untreated wastewater	RT-qPCR	(14.7 ± 2.2) × 10^3^ and (7.5 ± 2.8) × 10^3^ gc/L in the effluents.	[18]
	Japanese	Untreated wastewater	RT-qPCR	Influent (4.0 × 10^3^–8.2 × 10^4^ cg/L), treated wastewater (1.4 × 10^2^–2.5 × 10^3^ cg/L).	[35]
	Japanese	Untreated wastewater	RT-qPCR	1.2 × 10^3^–4.4 × 10^3^ gc/L.	[36]
	United States America	Untreated wastewater	RT-qPCR	3.0 × 10^4^ gc/L.	[37]
Emerging	Spain	Untreated wastewater	RT-qPCR	Of 5.22 and 5.99 log_10_ gc/L.	[38]
	Spain	Untreated wastewater	RT-qPCR	5.4 ± 0.2 log_10_ gc/L on average.	[38]
	Spain	Untreated wastewater	RT-qPCR	9 gc/mL rising to more than 20 gc/mL.	[39]
	Israel	Untreated wastewater	RT-qPCR	Ct of 33 to 33.6.	[40]
	Italy	Untreated wastewater	RT-qPCR	50% of the samples showed positive.	[41]
Underdeveloped	Mexico	Untreated wastewater	RT-qPCR	From 0.12 to 4 and 0.37–73 gc/mL.	[42]
	Turkey	Untreated wastewater	RT-qPCR	1.17 × 10^4^ y 4.02 × 10^4^ gc/L.	[43]
	Ecuador	Urban streams with low sanitation	RT-qPCR	2.84 × 10^5^ to 3.19 × 10^6^ and 2.07 × 10^5^ to 2.23 × 10^6^ gc/L.	[44]
	Ecuador	Lagoon systems	PCR	In GEN N1 36.44, GEN N2 38.99; GEN N1 36.80 GEN N2 38.72.	[28]

**Table 2:** Countries where evidence of SARS-CoV-2 was found in wastewater and confirmed cases of COVID-19 and world ranking.

Economic Status	Confirmed Cases	World Ranking Confirmed Cases	Country	Cases Number/100,000 Inhabitants’ Ratio
Developed	8,118,400	15	Netherlands	10,754
	27,124,689	5 *	Germany	4542
	85,007,630	1 *	United States of America	10,577
	7,719,719	16	Australia	137
	29,114,200	4 *	France	9286
	921,566	52	United Arab Emirates	6931
	4,127,625	29	China	8
	9,108,323	14	Japan	756
	12,551,142	11	Spain	9556
Emerging	4,216,009	27	Israel	10,224
	17,773,764	9 *	Italy	7316
	5,843,190	21	Mexico	2219
Underdeveloped	15,085,742	10 *	Turkey	6872
	891,064	56	Ecuador	2764

Elaborated with data from World Health Organization [9], as of 17 June 2022. * Top ten ranked worldwide.

**Table 3:** Shows the data on the positive cases of COVID-19 in Mexico and the information on the existing treatment plants with the relevant data on their operation.

No.	States	Accumulated Positive Cases	Estimated Assets	No. Plants	Installed Capacity (L^−1^/s) (to)	Treated Flow (L^−1^/s) (b)	% Treated (a)/(b)
1	Ciudad de México	621,287	9156	29	5604.50	2451.50	43.74
2	Estado de México	237,961	2526	131	9744.70	6400.10	65.68
3	Guanajuato	129,001	774	64	7560.80	5221.20	69.06
4	Nuevo León	120,840	721	55	16,157.00	12,590.40	77.93
5	Jalisco	83,685	660	122	15,245.20	12,346.20	80.98
6	Puebla	80,504	974	85	3516.90	3592.50	102.15
7	Sonora	71,456	540	109	7394.10	6115.90	82.71
8	Coahuila	67,231	253	26	5680.00	4516.00	79.51
9	Queretaro	66,253	1072	51	2449.40	1892.40	77.26
10	Tabasco	62,195	885	99	2969.90	2665.00	89.73
11	San Luis Potosi	61,150	572	40	2572.70	2101.00	81.67
12	Veracruz	58,559	391	108	7014.80	4711.90	67.17
13	Tamaulipas	55,239	352	47	7369.20	4096.40	55.59
14	Chihuahua	48,596	1099	185	10,263.10	7031.70	68.51
15	Baja California	46,969	280	Four. Five	7882.60	5977.80	75.84
16	Michoacan	45,936	410	46	4145.50	3175.40	76.6
17	Oaxaca	44,639	373	76	1817.60	1291.20	71.04
18	Guerrero	38,373	500	67	4428.30	3755.50	84.81
19	Hidalgo	37,259	409	56	23,826.80	22,133.90	92.89
22	Sinaloa	36,821	406	279	6496.70	5837.20	89.85
21	Yucatan	35,856	576	28	448.70	231.50	51.59
22	Durango	32,765	355	220	4638.70	3496.10	75.37
23	Morelos	30,996	459	52	2769.70	1276.40	46.08
24	Zacatecas	29,300	252	65	2012.40	1616.00	80.3
25	Baja California Sur	29,081	616	31	2051.30	1626.50	79.29
26	Aguascalientes	25,341	273	135	4840.00	2982.70	61.63
27	Quintana Roo	21,783	405	31	2685.00	1780.20	66.3
28	Tlaxcala	18,954	197	55	1481.80	1049.60	70.83
29	Nayarit	114,283	137	70	3493.80	2510.30	71.85
30	Colima	7601	102	82	2434.90	1739.80	71.45
31	Chiapas	6574	99	3. 4	2001.20	1343.60	67.14
32	Campeche	6016	67	17	155.00	142.80	92.13
		2,372,504	25,891	2540	181,152	137,699	

Source: Official figures from the Ministry of Health of 11 April 2021 [11] and National inventory of municipal drinking water and wastewater treatment plants in operation [69].

## Data Availability

The data presented in this study are available on request from the corresponding authors.
